# Ultralow voltage operation of biologically assembled all carbon nanotube nanomesh transistors with ion**-**gel gate dielectrics

**DOI:** 10.1038/s41598-017-06000-w

**Published:** 2017-07-20

**Authors:** Hye-Hyeon Byeon, Kein Kim, Woong Kim, Hyunjung Yi

**Affiliations:** 1 Post-Silicon Semiconductor Institute, Korea Institute of Science and Technology, Seoul, 02792 Republic of Korea; 2 Department of Nano Semiconductor Engineering, Korea University, Seoul, 02841 Republic of Korea; 3 Department of Materials Science and Engineering, Korea University, Seoul, 02841 Republic of Korea

## Abstract

The demonstration of field-effect transistors (FETs) based entirely on single-walled carbon nanotubes (SWNTs) would enable the fabrication of high-on-current, flexible, transparent and stretchable devices owing to the excellent electrical, optical, and mechanical properties of SWNTs. Fabricating all-SWNT-based FETs *via* simple solution process, at room temperature and without using lithography and vacuum process could further broaden the applicability of all-SWNT-FETs. In this work, we report on biologically assembled all SWNT-based transistors and demonstrate that ion-gel-gated network structures of unsorted SWNTs assembled using a biological template material enabled operation of SWNT-based transistors at a very low voltage. The compatibility of the biologically assembled SWNT networks with ion gel dielectrics and the large capacitance of both the three-dimensional channel networks and the ion gel allowed an ultralow operation voltage. The all-SWNT-based FETs showed an *I*_*on*_ */I*_*off*_ value of >10^2^, an on-current density per channel width of 2.16 × 10^−4^ A/mm at V_DS_ = 0.4 V, and a field-effect hole mobility of 1.12 cm^2^/V · s in addition to the low operation voltage of <−0.5 V. We envision that our work suggests a solution-based simple and low-cost approach to realizing all-carbon-based FETs for low voltage operation and flexible applications.

## Introduction

Single-walled carbon nanotubes (SWNTs) are one of the most promising electronic materials for high-performance field-effect transistors (FETs)^1, 2^. Field effect transistors (FETs) based entirely on carbon nanotubes (CNTs) have several advantages such as simple device design, improved contact at the channel-to source(S)/drain(D) interface, good optical transmittance and excellent mechanical flexibility^3–9^. The use of CNTs as electrodes instead of metallic electrodes provides not only mechanical flexibility and transparency but also good electrical contacts to an CNT channel due to ideally the similar work function of the CNT electrodes and channel^7, 10^. Improved electrical contact at the channel-to-S/D interface results in a small contact resistance, which could lead to high on-current^3^. Various approaches such as transferring, printing and self-assembly process have been developed to fabricate high performance CNT-based transistors^4, 5, 7–9, 11–16^. Many of the high-performance all CNT-based FETs have been demonstrated by employing semiconducting-enriched CNTs for the semiconducting channel^3, 4, 6, 8, 9^. Recently, unsorted CNTs have been also successfully employed for the fabrication of high-performance all CNT-based FETs showing *I*_*on*_ */I*_*off*_ > 10^5^ by controlling the network density of CNT channels around the percolating threshold^5, 16^. However, previously reported approaches require appropriate surface treatments of CNTs such as acid treatment and heat treatments to enhance electrical conductivity of CNTs or remove residual surfactants. Fabricating all-CNT-based FETs *via* simple solution process, at room temperature and without using lithography and vacuum process could further broaden the applicability of all CNT-based FETs. Previously, our group showed that aSWNT-network film could be successfully assembled in an aqueous solution using a biological template material under a hydrodynamic process, such as dialysis, to produce so called a SWNT nanomesh^17^. Such hydrodynamic process produced a free-standing SNWT-nanomesh film by releasing the nanomesh from the dialysis membrane^17^. Simple transfer of the free-standing nanomesh of unsorted SWNTs (U-SWNTs) using pre-patterned mask successfully produced channels for FETs^17, 18^. In this scheme, no chemical or heat treatment is required either to remove surfactants used for dispersing CNTs or to dissolve the supporting structure onto which the CNT film was deposited. However, the utilization of oxide gate dielectric layers such as SiO_2_ and HfO_2_ in previous works limited the operation voltage and/or required the vacuum process. Moreover, only metallic electrodes were employed and thus the possibility of fabricating all SWNT-nanomesh FET has not been demonstrated. As a proof-of-concept for fabricating all SWNT-FET *via* simple solution process at room temperature, ion-gel could be employed as gate dielectric since polymer/ionic liquid composite gels are attractive dielectric materials because of their high specific capacitance, excellent ionic conductivity, printability, and flexibility^11, 12, 15, 19–23^.

Here, we report on biologically assembled all SWNT-based transistors and demonstrate that the ion gel-gated all nanomesh-FETs operate at ultralow saturation voltages and show decent *I*_*ON*_/*I*_*OFF*_ and on-current density values. Network structures of unsorted SWNTs with tunable sheet resistance were assembled using a biological template material and employed for contact electrodes and channels depending on their resistance. The ion-gel dielectric layer was compatible with the biologically assembled SWNT networks in terms of wettability and chemical stability and the ion-gel-gated SWNT channels exhibited a very high total capacitance. The three-dimensional network structures of the SWNT channels and their good wettability with the ion gel were responsible for the high total capacitance, thus enabling transistor operation at ultralow saturation voltages. We also show that an amine-rich polymer layer can be used to further shift the threshold voltage of the SWNT channel. Moreover, using SWNTs-nanomesh as S/D electrodes to contact nanomesh channels was found to increase the on-current value about 15 fold on average compared to using Au electrodes. The transistors based entirely on the SWNTs showed a *I*_*ON*_/*I*_*OFF*_ value of >10^2^, an on-current value per channel width of 2.16 × 10^−4^ *A*/*mm* at *V*_*DS*_ = 0.4 *V*, a field-effect hole mobility of 1.12 *cm* ^2^/*V* · *s*, and an ultralow operation voltage of <−0.5 V. We envision that our simple and low-cost method to fabricate all-SWNT-based high-performance FETs will provide a valuable route to future electronic devices that require low-voltage operation, mechanical flexibility and transparency.

## Results

### Ion-gel-gating of biologically assembled SWNT network channel-based FETs

**Figure 1:**
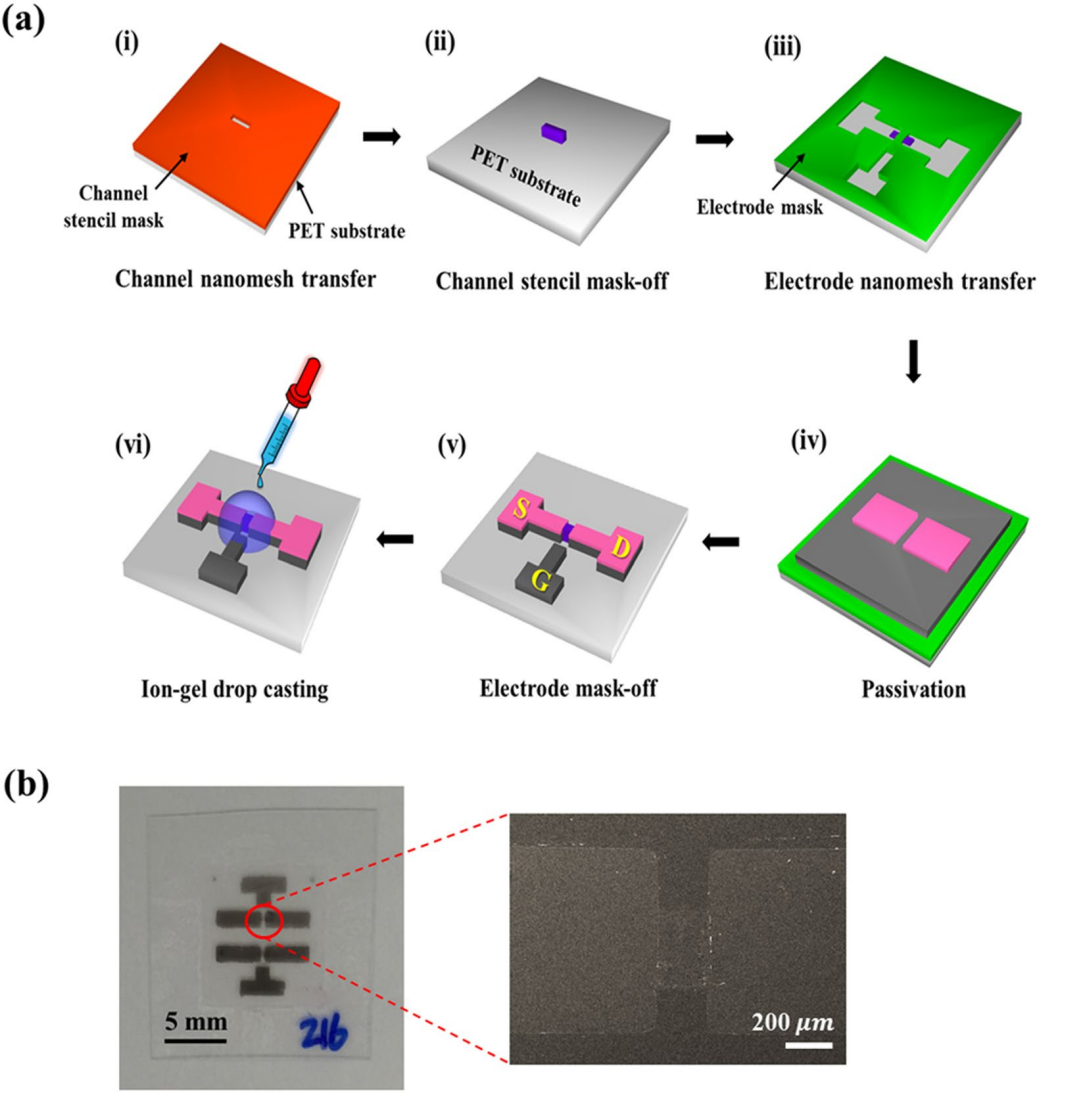
Fabrication of the ion-gel-gated all-SWNT-nanomesh-based FET. (**a**) Processes used to fabricate the all-SWNT-based ion-gel-gated FET. Different SWNTs-nanomesh compositions were used to fabricate the channels and the electrodes (source (S), drain (D), and gate (G)). (**b**) Photograph and scanning electron micrograph of the all-SWNT-based FET. The channel length and width were 200 μm and 400 μm, respectively.

Figure 1 schematically illustrates the process to fabricate the all-SWNT-based ion-gel-gated FETs. The samples were fabricated on a transparent and flexible PET substrate. A nanomesh of SWNTs and M13 phage assembled in an aqueous solution were transferred using a pre-patterned stencil mask to form channels and source, drain, and gate electrodes. The M13 phage used is a filamentous biological material that has strong binding affinity toward SWNTs on its body surface and the SWNTs and the M13 phage bind each other along their lengths^17^. The ion-gel solution was drop-cast onto the device with the channel region and all of the electrodes having been patterned, and dried to form a gate dielectric layer. The source and drain electrodes were passivated using the stencil mask before forming the ion-gel film to minimize leakage current. A photograph of the final device and a scanning electron micrograph of the channel region (circled red in the photograph) are shown in Fig. 1b. It is noted that the channel is transparent (Fig. S1). The length and width of the channel were designed to be 200 μm and 400 μm, respectively, and the SEM image confirmed that the size of the channel was consistent with the mask design.

**Figure 2:**
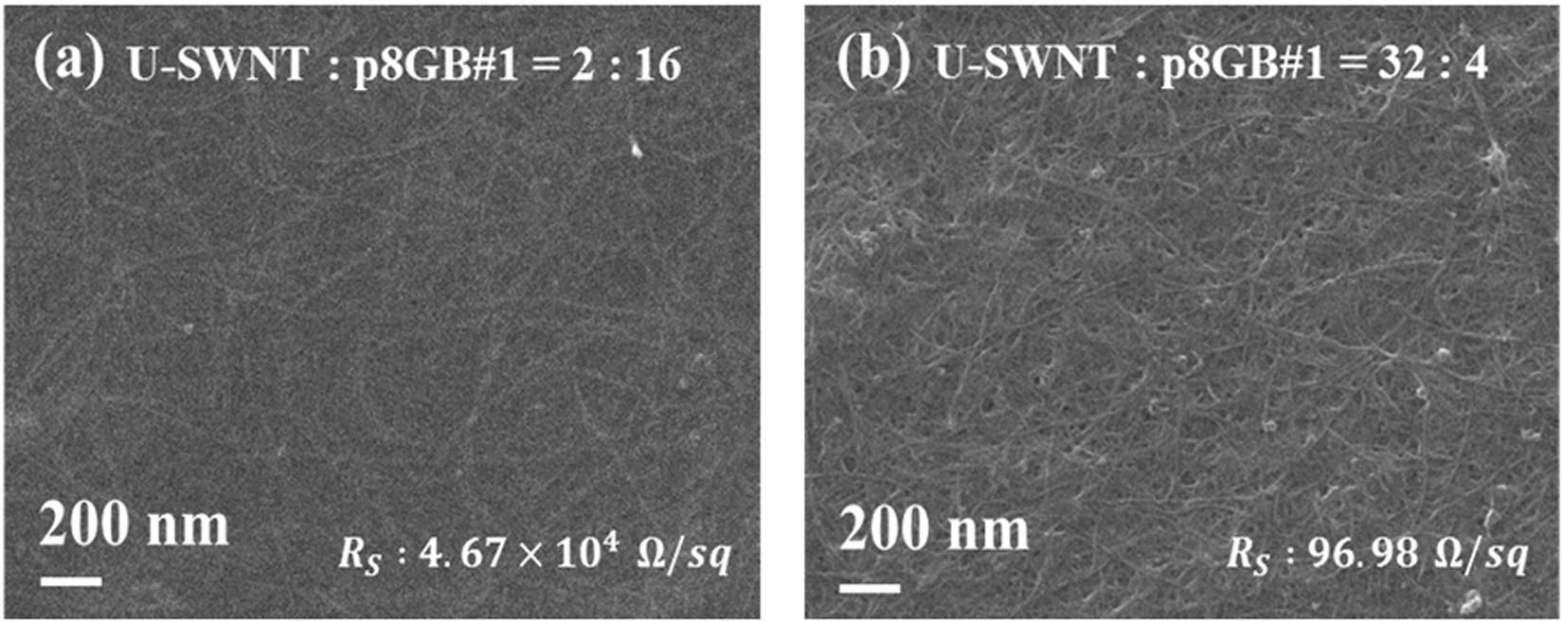
Characteristics of the channel and electrode nanomeshes. Scanning electron micrographs and the sheet resistance values of the nanomesh used for (**a**) the channel and (**b**) the S/D/G electrodes.

To fabricate the all-SWNT-based FETs, we chose U-SWNT:p8GB#1 molar ratios of 2:16 and 32:4 for the channel and S/D electrodes, respectively. Low content of the SWNTs in the nanomesh was previously shown to be essential to provide the channel with high on/off ratio, while high concentration was required to achieve low electrical resistance of the electrodes^17, 18^. A scanning electron micrograph of each nanomesh is shown in Fig. 2 along with its sheet resistance (*R*_*S*_) value. The sharp and high-contrast lines correspond to the SWNTs (Fig. S2). Note that the insulating and soft biological material is not clearly visualized in the SEM image. The SEM images clearly show the SWNTs to be well dispersed in the nanomesh film. The SWNTs are less dense in the nanomesh channel than those in the nanomesh electrode. The sheet resistances of the channel nanomesh and electrode nanomesh were 4.67 × 10^4^ and 96.98 Ω/sq, respectively. The higher resistance of the channel nanomesh was attributed mainly to its lower density of SWNTs dispersed in insulating biological template material. The thickness of the channel nanomesh and electrode nanomesh were 300 nm and 800 nm, respectively. The large thickness of the nanomesh suggests the three-dimensional nature of the SWNT network.

**Figure 3:**
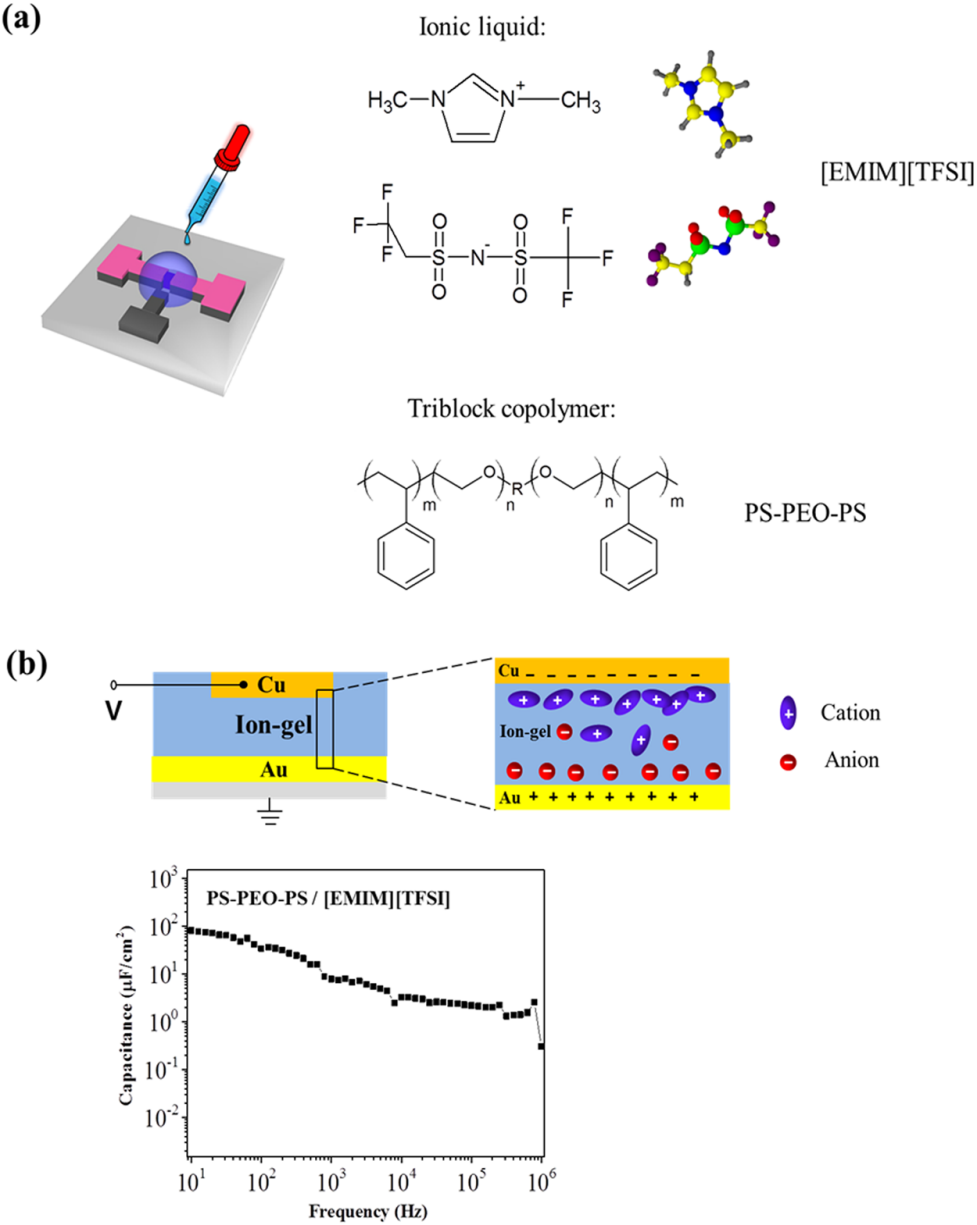
Characteristics of the ion-gel gate dielectrics. (**a**) Molecular structures of the ion gel components: the PS-PEO-PS triblock copolymer and the [EMIM][TFSI] ionic liquid (into which the triblock polymer was dissolved). (**b**) Frequency dependence of the specific capacitance of the ion-gel film. Schematics of the tested Au/ion gel/Cu device structure and of ion distribution are also shown. The composition of the ion - gel was [EMIM][TFSI]:PS-PEO-PS = 96:4 (% w/v).

The structure of the ion gel-based gate dielectric employed in this study is shown in Fig. 3a. A triblock copolymer, poly(styrene-block-ethylene oxide-block-styrene) (PS-PEO-PS), was dissolved in an ionic liquid, 1-ethyl-3-methylimidazolium bis(trifluoromethylsulfonyl)imide ([EMIM][TFSI]). The PS-PEO-PS triblock copolymers formed well-defined physical gels through non-covalent association of the PS blocks^22, 24, 25^. An ion-gel film was readily formed by drop-casting an acetonitrile solution containing the [EMIM][TFSI] ionic liquid and PS-PEO-PS copolymer onto target substrates. We prepared two ion gel solutions containing different concentration of PS-PEO-PS triblock copolymer, 4% w/v and 7% w/v. The capacitance of the ion gel was measured in metal-insulator-semiconductor (MIS) structures as shown in Fig. 3b. It was measured as a function of frequency (from 10 to 10^6^ Hz) using an electrochemical impedance analyzer (Versastat, Princeton Applied Research). The gel thickness was approximately 300 μm. The low concentration of PS-PEO-PS triblock copolymer resulted in a higher capacitance, presumably due to the more effective formation of electrical double layer by the ionic liquid (Fig. S3). The capacitance of the ion gel layer having PS-PEO-PS at 4% w/v was measured to be ~81.90 μF *cm* ^−2^ at 10 Hz. This capacitance value was comparable to or slightly higher than previously reported values^22, 24, 25^, suggesting the high quality of the synthesized ion gel.

**Figure 4:**
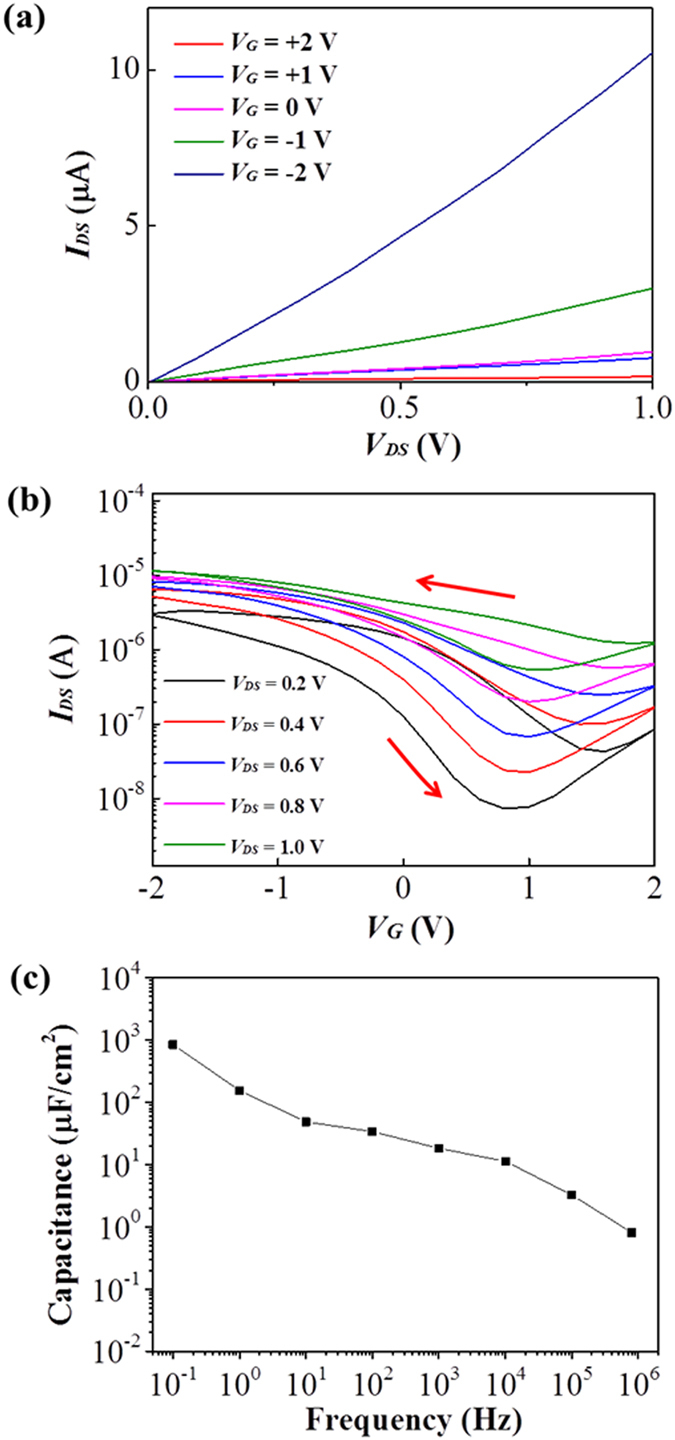
Characteristics of the ion-gel-gated nanomesh-based FETs with Au electrodes. (**a**) The output characteristics and (**b**) transfer characteristics of the nanomesh-based ion-gel-gated FET shown with hysteresis. The U-SWNT:p8GB#1 molar ratio of the channel was 2:16. The hysteresis is indicted by arrows. (**c**) Frequency dependence of the maximum capacitance for ion gel/nanomesh assembled at a U-SWNT:p8GB#1 molar ratio of 2:16.

The effect of the ion - gel-based dielectric layer on the performance of SWNT-FETs was investigated with metallic S/D/G electrodes (Au) first. The output characteristics (*I*_*DS*_ vs. *V*_*DS*_) and the transfer characteristics (*I*_*DS*_ vs. *V*_*G*_) of a representative ion-gel-gated FET from the 2:16 nanomesh channel with Au electrode are shown in Fig. 4a,b. The highest *I*_*ON*_/*I*_*OFF*_ value was found to be ~3.94 × 10^2^ at *V*_*DS*_ = 0.2 *V*. The *I*_*ON*_ and *I*_*OFF*_ values at this voltage were observed to be 2.90 × 10^−6^ A and 7.35 × 10^−9^ A, respectively. The threshold voltage, *V*_*th*_, was 0.2 V at *V*_*DS*_ = 0.2 *V* (Fig. S4). In our previous studies, the operation voltage of the local bottom-gated nanomesh FET with a HfO_2_ dielectric layer (0.44 μF *cm* ^−2^) was <5 V and this value was still a greatly improved one compared to the ~60 V value of the bulk SiO_2_ dielectric layer (0.012 μF *cm* ^−2^)-gated nanomesh FETs^17, 18^. The FET showed an anticlockwise hysteresis as indicated by arrows in Fig. 4b. The voltage difference between gate voltages needed to induce an average of the maximum and minimum drain current for the forward and reverse sweep directions was estimated to be 0.28 V at V_DS_ = 1.0 (Fig. S5). The noticeable hysteresis is presumably due to the adsorbed water and oxygen molecules by the hydrophilic biological material in contact with the SWNTs since the nanomesh was assembled in aqueous solution^26, 27^.

The ultralow threshold voltage of ~0.2 V of the ion-gel-gated nanomesh FETs suggested that the capacitance of the nanomesh channel of the biologically assembled SWNTs was large enough to effectively exploit the large capacitance of the ion-gel dielectric and that the nanomesh channel was highly compatible with the ion-gel dielectric. To confirm this, the total capacitance of the nanomesh-based ion-gel gated transistor was measured and shown in Fig. 4c. The total capacitance was measured to be ~48.70 μF *cm* ^−2^ at 10 Hz (Fig. 4c) and the capacitance of the SWNT nanomesh was calculated to be ~120.14 μF *cm* ^−2^ (see Method section), confirming the large capacitance of the SWNT nanomesh channel. The wetting angle of the ion-gel on the nanomesh channel was measured to be 35.72 degree (Fig. S6), implying the good wettability of the ion-gel with the nanomesh. In order to examine the chemical compatibility of the nanomesh in contact with the ion-gel, the on-current and off-current levels of the ion-gel gated nanomesh-FET was compared with those obtained from devices stored in ambient condition for ten days (Table S1). The current levels did not notably change, suggesting the chemical compatibility of the nanomesh with the ion-gel. The three-dimensional network structure of the nanomesh channel and the compatibility with the ion gel were presumably the main factors responsible for the large channel capacitance and thus enabled highly effective gating of the SWNT-FETs. It is noted that the *I*_*ON*_/*I*_*OFF*_ value of the ion-gel-gated FET was also much higher than that of the local bottom-gated FETs or back-gated FETs^17, 18^.

### Shift of the threshold voltage

**Figure 5:**
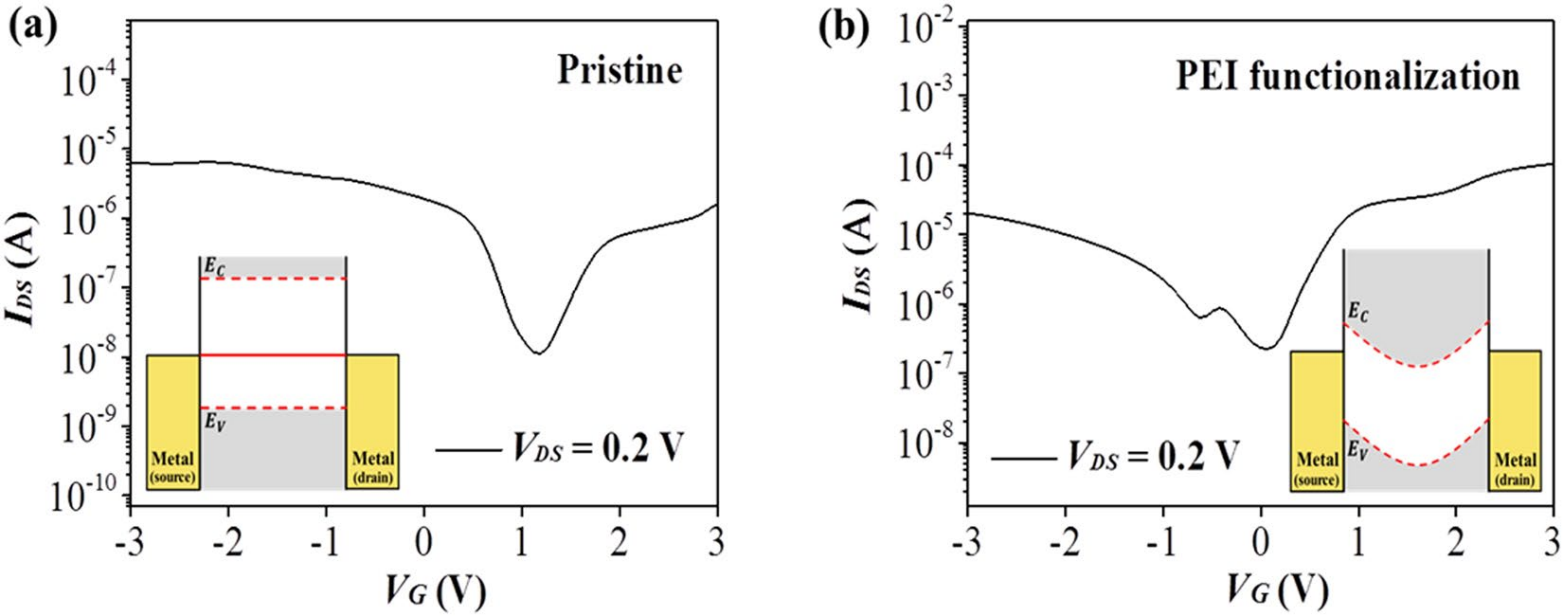
The shift of the threshold voltage of the ion-gel-gated nanomesh-based FETs using a PEI layer. Transfer characteristics of the nanomesh-based ion-gel-gated FETs (**a**) before and (**b**) after PEI functionalization. The U-SWNT:p8GB#1 molar ratio of the channel nanomesh was 1:8. The inset depicts a qualitative band diagram of the device before and after PEI functionalization.

The threshold voltage was further shifted by introducing an electron donating polymer layer into the SWNT nanomesh channel. Polyethyleneimine (PEI) was selected in this work since a PEI layer has been demonstrated to serve as an electron-donating agent for SWNTs. The PEI used in experiments was a highly branched polymer with an average molecular weight of about 800, with about 25%, 50%, and 25% of its amino groups being primary, secondary, and tertiary amines, respectively^28^. Figure 5 compares the typical transfer characteristics of the nanomesh-FETs with Au electrodes at *V*_*DS*_ = 0.2 *V* before and after PEI functionalization of the nanomesh channel. The transfer curves clearly showed that the threshold voltage was shifted toward the more negative voltage direction as a result of the functionalization, with a fully coated nanotube showing a shift of approximately −1.40 V. The shift in the threshold voltage of the transistors indicated that the transition of the majority carrier type from hole to electron became more facile, confirming the electron donation effect of the PEI layer^28, 29^. Also note that the electron drive current, *I*_*ON*_, improved by one order of magnitude. These results could be also attributed to the reduced Schottky barrier (SB) between the electrodes and the semiconducting channels *via* interface dipole moment enabled by the electron transfer from the dopants to the channels^29, 30^. The insets of Fig. 5 schematically depict the band diagram of the SWNT-FETs before (pristine) and after PEI functionalization of the device. The insets show the reduced SB for electron injection, an increased SB for hole injection, and band bending due to the electrons transferred from the dopants to the SWNTs. These results highlight the compatibility of the SWNT nanomesh with a variety of functional polymers that has been utilized for tuning bare SWNTs.

### Ultralow voltage operation of all-SWNT-based FETs

**Figure 6:**
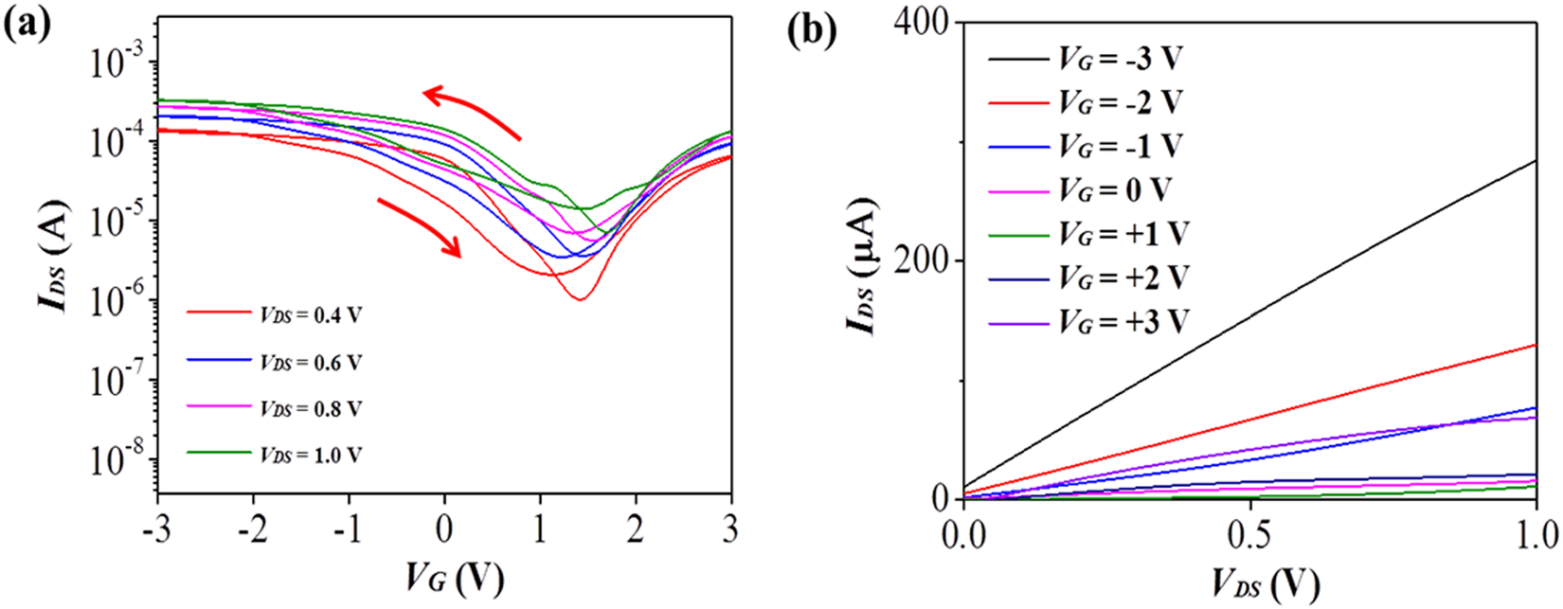
Characteristics of the ion-gel-gated all-nanomesh-based FET. (**a**) The transfer characteristics shown with hysteresis and (**b**) output characteristics of the nanomesh electrode-based ion gel-gated FET. The channel length was 200 *μm* and the channel width was 400 *μm*. The gate voltage was swept at a rate of 70 *mVs* ^−1^. The hysteresis is indicated by arrows.

Ion-gel-gated FETs based on entirely on the nanomeshes were fabricated according to the processes illustrated in Fig. 1. Nanomeshes with different compositions were employed. In particular, a highly conductive nanomesh (with a U-SWNT:p8GB#1 molar ratio of 32:4) was employed as the S/D/G electrodes. The transfer characteristics and output characteristics of a representative all-nanomesh-based ion-gel-gated FET are shown in Fig. 6a,b. The average *I*_*on*_/*I*_*off*_ value from three different devices was found to be ~(1.60 ± 1.06) × 10^2^ at *V*_*DS*_ = 0.4 *V*. The average *I*_*on*_ and *I*_*off*_ values were observed to be (8.64 ± 4) × 10^−5^ *A* and (7.4 ± 3.3) × 10^−7^ *A*, respectively. The on-current here was larger than that for the Au electrodes at V_DS_ = 0.4 by ~15 folds on average (Table S2) while exhibiting a similar *I*_*on*_/*I*_*off*_ value. The large increase in the on-current is ascribed to the improved contact resistance by the SWNT-nanomesh S/D electrodes compared to the Au electrodes^3, 31^. Since the resistance of the nanomesh channel is relatively low due to the relatively high network density (transmittance at 550 nm is ~80%, Fig. S1) compared to sparse-SWNT channel used for high-performance all CNT FETs^5, 16^, the reduction of the contact resistance could readily increase the on-current. The increased off-current level is presumably due to the increased leakage current since it is possible that the thick SWNT network electrodes could not be completely blocked by the passivation. The hole mobility, *μ*_*h*_, was estimated to be 1.12 *cm* ^2^/*Vs* at *V*_*DS*_ = 0.4 *V* (Supplementary equation 1). This mobility value is much lower than the one of the state-of-the-art CNT FETs (~1 057 *cm* ^2^/*Vs*)^5^ but at the same order as printed CNT FETs using unsorted CNTs^16^. The low mobility of our device could be ascribed to the phage present in the channel as in the hybrid CNT channel^32, 33^. The high on-current density per channel width of 2.16 × 10^−4^ A/mm even at the low mobility could be ascribed to the better S/D contact with channel, high SWNT network density of the nanomesh channel and the high gate capacitance^3, 5^. The threshold voltage, *V*_*th*_, was estimated to be ~0.44 V (Fig. S7). The all-SWNT-FET also showed an anticlockwise hysteresis as indicated by arrows. The larger hysteresis (Fig. S8) compared to Fig. 4(b) could be due to the more phage in the nanomesh electrodes in contact with the channel and/or the wider scanning voltage range^3^.

**Figure 7:**
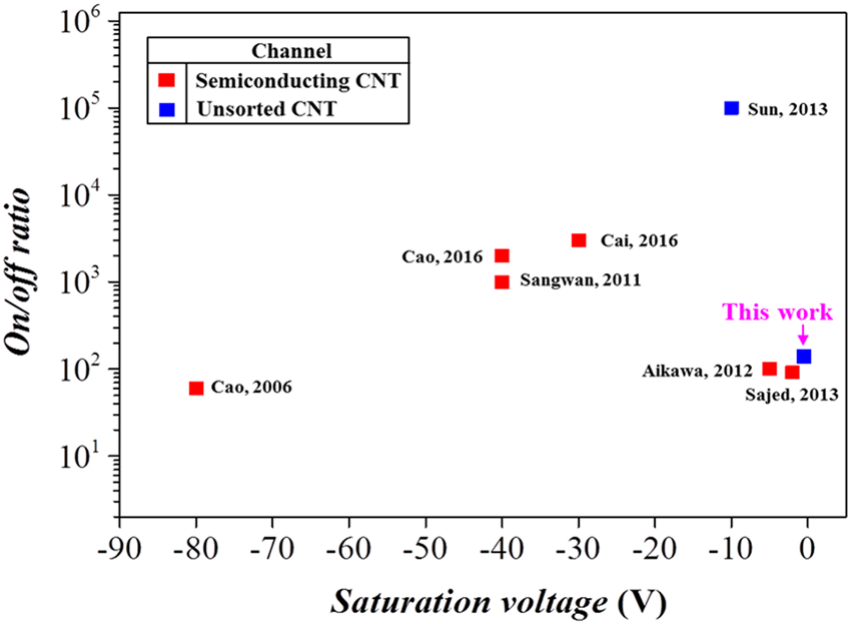
Comparison of the performance of the biologically assembled, ion-gel-gated all-SWNT-based FETs reported in this work with other all-CNT-based FETs fabricated using other approaches.

Figure 7 summarizes a comparison of the performance of the all SWNT nanomesh-based FETs with other all-CNT-based FETs in terms of the *I*_*on*_/*I*_*off*_ value and the saturation voltage^3–6, 8, 9, 15^. Due to the limited number of reported works on FETs entirely based on unsorted CNTs, devices fabricated using semiconducting-enriched channels have been also included. The saturation voltage was estimated from the inflection point in the transfer characteristics plotted in logarithmic scale. Relative to these other FETs, the device reported in this work exhibited the lowest saturation voltage and a decent *I*_*on*_/*I*_*off*_ value. It is worth noting that our device was fabricated without relying on lithographical method and chemical and heat treatment.

## Discussion

We have demonstrated all-SWNT-nanomesh-based FETs by employing SWNT nanomeshes of different compositions for channels and electrodes. The ion gel-gated nanomesh-FETs exhibited an ultralow saturation voltage (|V_sat_| < |−0.5 V|). The biologically assembled nanomesh of SWNTs possessed a very high specific capacitance, being comparable to that of ion-gel dielectrics. Thus, the field effect was effectively applied onto the channel of the FETs and accordingly enabled ultralow voltage operation. The employment of SWNT-nanomesh S/D electrodes instead of Au electrodes increased the on-current by ~15 folds on average, producing on-current density per channel width of 2.16 × 10^−4^ A/mm. The all-SWNT-based FETs showed an *I*_*ON*_/*I*_*OFF*_ value of >10^2^ and a low operating voltage of less than −0.5 V. The solution-based and room-temperature method to fabricate all-SWNT-based FETs will provide a facile route to realizing electronic devices that require low-voltage operation, mechanical flexibility and transparency. Other types of gate dielectric materials could be further explored for high-speed or low-power device applications.

## Methods

### Preparation of the ion gel dielectric film

A poly(styrene-block-ethylene oxide-block-styrene) (PS-PEO-PS) triblock copolymer was purchased from Polymer Source, Inc. The number average molecular weight and polydispersity index of the copolymer was 57 kg/mol (Mn(PS) = 10 kg/mol and Mn(PEO) = 37 kg/mol) and 1.20, respectively. An ionic-liquid-based polymer electrolyte was fabricated by adding 63.3 mg (114.4 mg) of the copolymer into 1 mL of [EMIM][TFSI] (C-TRI) for 4% w/v (7% w/v). The mixture was dissolved in 2 mL of acetonitrile and magnetically stirred for 12 h in a nitrogen atmosphere using a Schlenk line. The acetonitrile was removed by applying a temperature of 70 °C for 1 h.

### Fabrication of the all-nanomesh-based ion-gel-gated FETs

The nanomesh made up of single-walled carbon nanotubes (SWNTs) was assembled according to the previously reported method^17^. Briefly, an as-received unsorted SWNT solution (Superpure, from NanoIntegris Inc.) was surfactant-exchanged by a sodium cholate solution (anionic surfactant, 2% w/v in deionized water). The SWNTs stabilized by the sodium cholate surfactant were mixed with the p8GB#1 M13 phage showing strong binding affinity toward SWNTs on its body surface. Various SWNT:p8GB#1 molar ratios were used, in particular 32:4 and 2:16 for the assembly of source/drain electrodes and the channel, respectively. The mixed solution was then put into a dialysis membrane and dialyzed against deionized water with frequent changing of the dialyzing solution. After about 24 h, the dialyzed membrane bag was taken into a container filled with water and then the dialysis membrane was removed to produce a large-area nanomesh film floating in water. The nanomesh made using 2:16 SWNT:p8GB#1 molar ratio was transferred onto a transparent flexible poly(ethyleneterephthalate) (PET) substrate using a pre-patterned stencil mask. The transferred nanomesh was left to dry in air, and then the stencil mask was lifted off to produce channels. Then, an additional nanomesh made using 32:4 SWNT:p8GB#1 molar ratio was deposited using the stencil mask to form source/drain (S/D) contact electrodes and the gate (G) electrode. The length and width of the channel were set to 200 μm and 400 μm, respectively. The surface of the S/D electrodes was passivated using a cyanoacrylate adhesive. For the PEI functionalized FETs, 5 μL of the PEI solution (average molecular weight ~800, density ~1.050 g/mL, Sigma-Aldrich) was drop-casted onto the SWNTs channel and air-dried for 24 h. Then, the ion-gel solution was drop-cast on the channel and the exposed gate region, followed by being baked at 70 °C for 5 min.

### Characterizations

The sheet resistance of the nanomesh was measured using the van der Pauw method (Hall effect measurement system, HMS-3000). The nanostructures of the nanomesh were examined using scanning electron microscopy (SEM) and transmission electron microscopy (TEM). For SEM, samples were imaged in their native condition (no conductive coating applied) at an acceleration voltage of 20 kV using a JSM-6500F field emission scanning electron microscope. For TEM, the nanomeshes was transferred to a TEM grid (QUANTIFOIL 2 μm circular holes, TedPella Inc.) and dried at room temperature. TEM was performed using a Quantum 966 of FEI Titan, operated at 300 kV. For the contact angle measurement of the ion-gel on the nanomesh channel (2:16), 4 μL of the ion-gel was dropped onto the nanomesh film in ambient condition and analyzed using contact angle measurement system (Phoenix 300, SEO Co. Ltd.). The capacitance of the ion gel film and the ion gel-gated transistors was measured using an electrochemical impedance analyzer (Versastat, Princeton Applied Research). Impedance measurements were performed by using a two-electrode system. For the ion-gel-gated FETs, the source electrode was designated as working electrode (WE) and the gate electrode was designated as counter electrode (CE). Impedance spectra were recorded over a frequency range from 1 MHz to 0.1 Hz, at zero dc potential, with ac amplitude of 10 mV.

The transfer characteristics (the drain current (I_DS_) *vs*. gate voltage) of the ion-gel-gated transistors were measured by biasing from V_G_ = −3 V to 3 V at a scanning speed of 70 mV/s using an Agilent 4156 C at room temperature. The output characteristics (I_DS_ *vs*. the source-drain voltage (V_DS_)) were measured by scanning V_DS_ from 0 V to 1 V at a scanning speed of 70 mV/s.

### Calculation of the nanomesh capacitance

The ion-gel capacitance and the nanomesh (network-structured composite of the SWNTs and M13 phage) capacitance were connected in series. Therefore,

1 $$\frac{1}{{C}_{total}}=\frac{1}{{C}_{nanomesh}}+\frac{1}{{C}_{iongel}}$$

Note that, according to this equation, the component (i.e., nanomesh or ion gel) having the smaller capacitance dominates the overall capacitance, *C*_*total*_. However, when the nanomesh and ion gel have similar capacitance values, both values should be considered. The overall capacitance and the ion gel capacitance were measured to be ~48.70 μF/*cm* ^2^ and ~81.90 μF/*cm* ^2^ at 10 Hz, respectively. The nanomesh capacitance was estimated from these values using equation (1). The capacitance of the biologically assembled nanomesh with a U-SWNT:p8GB#1 molar ratio of 2:16 was calculated to be ~120.14 μF/*cm* ^2^.

## Electronic supplementary material

Supplementary information
