# Supplementary material for: Ultralow voltage operation of biologically assembled all carbon nanotube nanomesh transistors with ion-gel gate dielectrics
Source: Sci Rep. 2017 Jul 20;7:5981. doi: 10.1038/s41598-017-06000-w (PMC5519712; doi:10.1038/s41598-017-06000-w)
Supplement: Supplementary file 1 — Supplementary information [file 41598_2017_6000_MOESM1_ESM.pdf]

Supplementary information for

Ultralow voltage operation of biologically assembled all carbon nanotube nanomesh transistors with ion-gel gate dielectrics

Hye-Hyeon Byeon[1,2], Kein Kim[3], Woong Kim[3], Hyunjung Yi[1*]*

[1]Post-Silicon Semiconductor Institute, Korea Institute of Science and Technology, Seoul, 02792, Republic of Korea

[2]Department of Nano Semiconductor Engineering, Korea University, Seoul, 02841, Republic of Korea

[3]Department of Materials Science and Engineering, Korea University, 02841, Republic of Korea

[*] Address correspondence to hjungyi@kist.re.kr, woongkim@korea.ac.kr.

Supplementary Figures

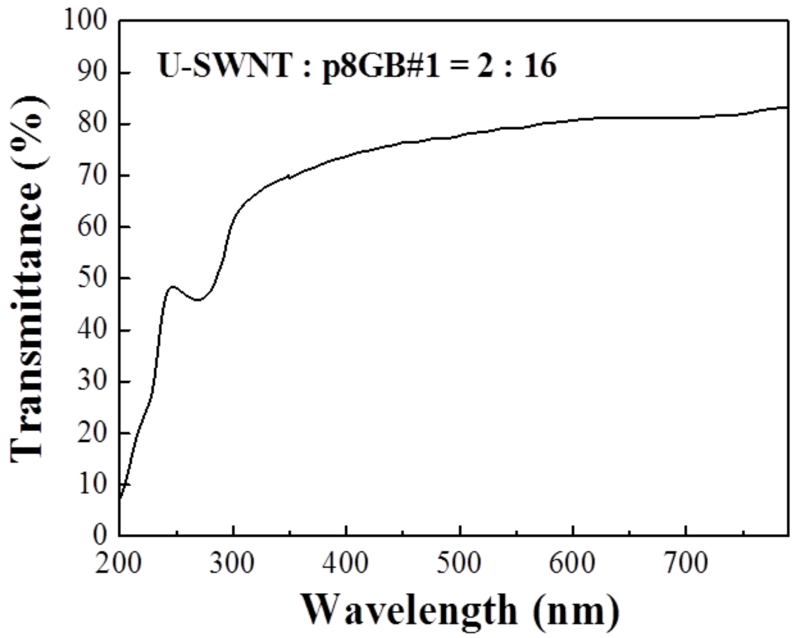

Figure S1. Optical transmission spectrum of the nanomesh, including the visible range. The U-SWNT:p8GB#1 molar ratio =2:16. The optical transmittance of the nanomesh at 550 nm was approximately 80 %.

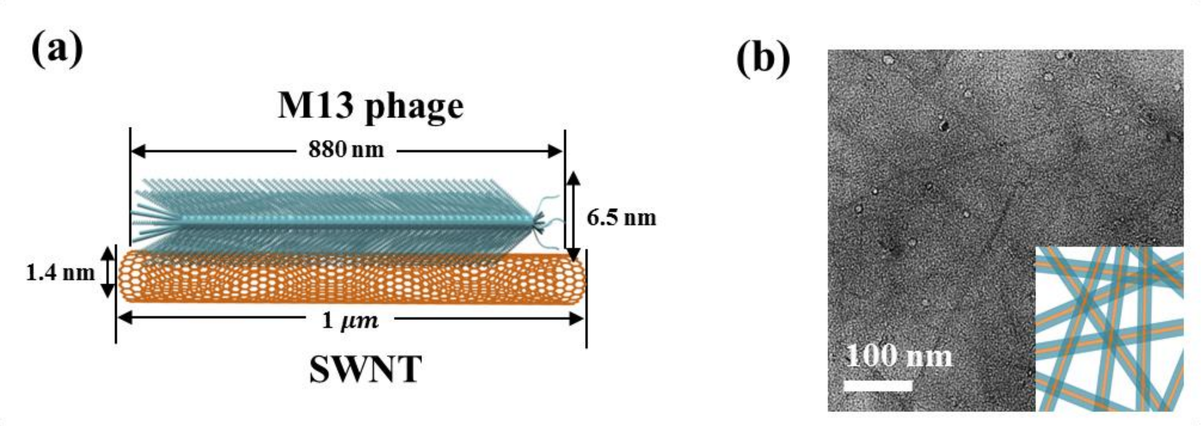

Figure S2. Components and the nanostructure of the nanomesh. (a) Binding scheme of the M13 phage and the SWNTs. Their respective dimensions are also shown. (b) Transmission electron micrograph of the nanomesh channel used in this study.

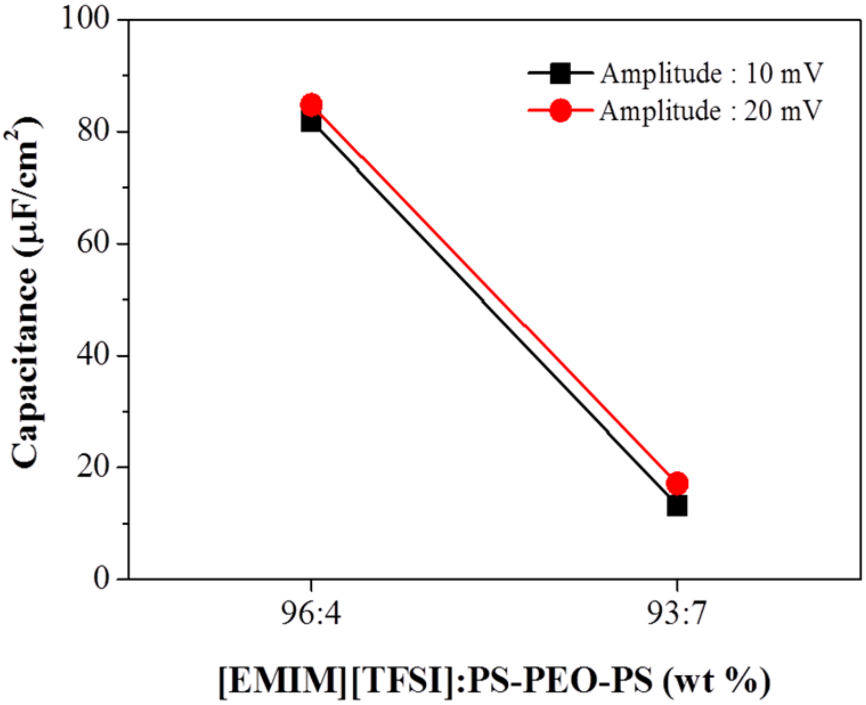

Figure S3. The dependence of the capacitance of the ion gel on its composition.

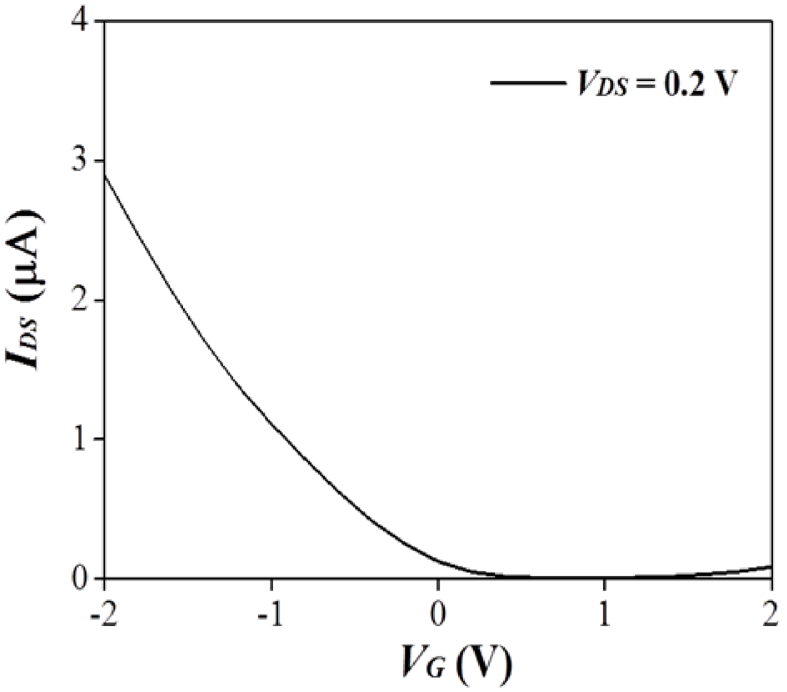

Figure S4. The transfer characteristics (I_{DS} vs. V_G, $V_{DS} = 0.2\,V$) of the ion-gel-gated nanomesh-based FET with Au S/D electrode shown in linear scale.

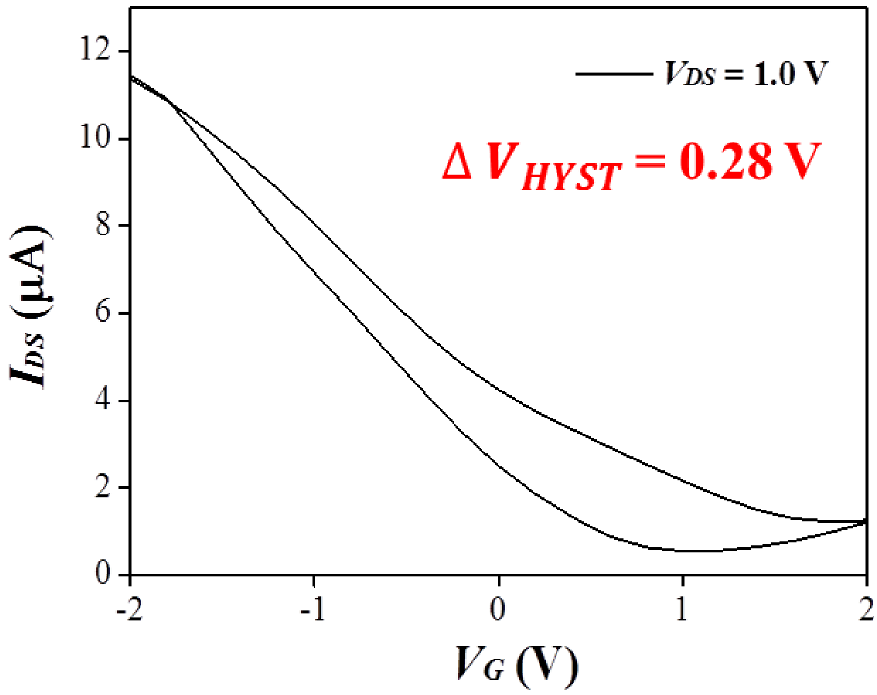

Figure S5. The hysteresis of the ion-gel-gated nanomesh-based FET with Au S/D electrode. ΔV_{HYST} is the voltage difference between gate voltages needed to induce an average of the maximum and minimum drain current for the forward and reverse sweep directions

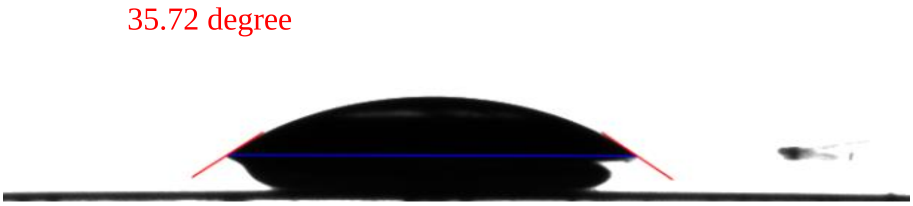

Figure S6. Contact angle measurement of the ion-gel on the channel nanomesh on PET substrate

$V_{DS} = 0.2\ V$	On-current (A)	Off-current (A)	I_{on}/I_{off}
As-fabricated	2.8973×10^{-6}	7.3457×10^{-9}	$3.94 \times\ 10^{2}$
After ten-days	1.8204×10^{-6}	5.5467×10^{-9}	$3.28 \times\ 10^{2}$

Table S1. Comparison of the current levels of the as-fabricated, ion gel-gated nanomesh-FETs with those measured after ten days.

(i) nanomesh channel (2:16) and Au S/D electrode, V_{DS}=0.4 V

Device	On-current (A)	Off-current (A)
#1	6.3672×10^{-6}	2.2502×10^{-8}
#2	5.6853×10^{-6}	1.7228×10^{-8}
#3	4.8532×10^{-6}	2.4452×10^{-8}
Average$\pm$Standard deviation	$(5.6352\pm0.62) \times 10^{-6}$	$(2.1394\pm0.31) \times 10^{-8}$

(ii) nanomesh channel (2:16) and nanomesh S/D electrode (32:4), V_{DS}=0.4 V

Device	On-current (A)	Off-current (A)
#1	1.3830×10^{-4}	9.9097×10^{-7}
#2	8.1022×10^{-5}	2.7091×10^{-7}
#3	3.9947×10^{-5}	9.5837×10^{-7}
Average$\pm$Standard deviation	$(8.642\pm4.0) \times 10^{-5}$	$(7.4008\pm3.32) \times 10^{-7}$

Table S2. Comparison of the on-current and off-current levels of ion-gel-gated nanomesh FETs that have Au as S/D electrodes with those having nanomesh electrode (32:4)

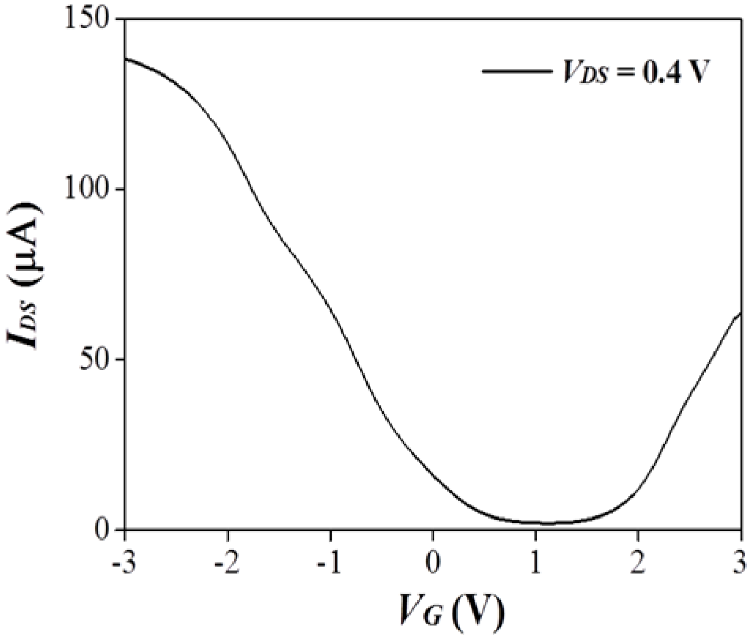

Figure S7. The transfer characteristics (I_{DS} vs. V_G, $V_{DS} = 0.4\,V$) of the ion-gel-gated all-nanomesh-based FET shown in linear scale.

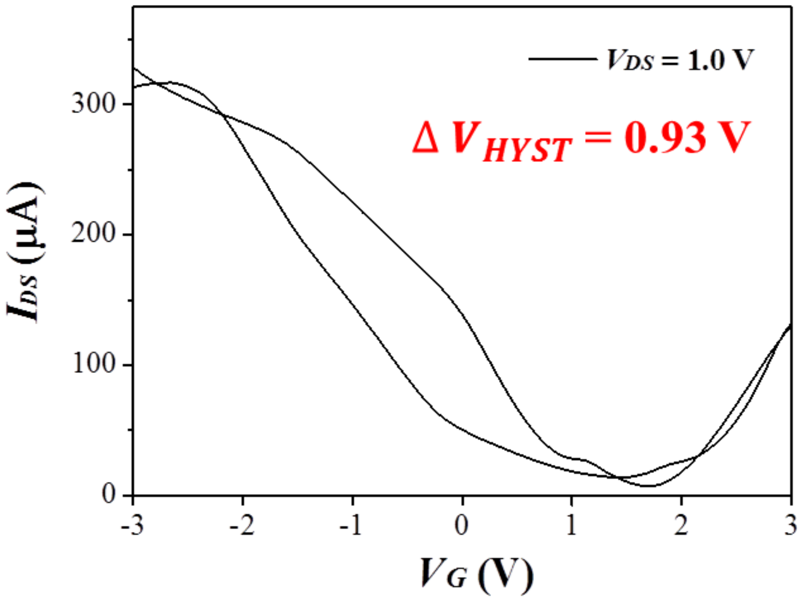

Figure S8. The hysteresis of the ion-gel-gated all-nanomesh-based FET.

Supplementary Equations

Calculation of the hole mobility of the ion gel-gated nanomesh-FET

The field-effect hole mobility of the ion gel-gated FETs can be calculated using the equation,

$$\mu_p = \left(\frac{dI_{DS}}{dV_G}\right) \times \frac{L}{W} \times \frac{1}{C_{total}V_{DS}} \qquad \cdots\cdots \ (1)$$

The L (200 μm) and W (400 μm) represent the channel length and width, respectively. I_{DS}, V_G and V_{DS} are the source-drain current, gate voltage and source-drain voltage, respectively. From these values, the hole mobility was estimated to be $\mu_h = 1.12\ cm^2/V\ s$ at $V_{DS} = 0.4\ V$.
